# Scientific Thinking About Legal Truth

**DOI:** 10.3389/fpsyg.2022.918282

**Published:** 2022-07-06

**Authors:** Gal Rosenzweig

**Affiliations:** Faculty of Law, Bar-Ilan University, Ramat Gan, Israel

**Keywords:** external validity, decision making, legal truth, scientific evidence, perception, discretion, eyewitness testimony, risk management

## Abstract

In the criminal process, the fact finders assess the validity of impressions reported by witnesses based on their perceptions and determine what has happened in reality. However, these impressions are not subject to any external validity check. The Innocence Project revealed the failure of this subjective method and showed how it can lead to innocent convictions. The legal literature has examined ways to manage the risk of mistakes, but these ways are inconsistent with the scientific understanding of the need for external validity measurements, suggesting the need for new ways of thinking about the legal search for truth and justice.

Criminal trials involve witness reports about a crime to the court or the jury members. The reports are based on various sources of information, such as the personal impressions of an eyewitness or an expert's conclusion drawn from forensic measurements. Fact finders (the judge or jury members) assess the validity of witness reports based on their subjective perception to determine what has happened in reality (Bell, 2013). Given the need to decide within a limited time and to meet many divergent goals, flexible discretion is crucial. The rules of evidence form the framework for allocating the risks of a mistake owing to the inherent uncertainty involved in the process (Summers, 1999). Although the fact finders check the validity of the witness testimonies and base their decisions on forensic evidence, there is no external measurement to validate the impressions fact finders form based on witness testimonies (Menashe, 2008). Even the validity assessment of scientific evidence is based on estimations because the legal system assumes that implementing a scientific norm proves the conclusion straightforwardly, ignoring the effects of the diverging assumptions needed for individualization on the strength and validity of the conclusion (Kaye, 2010).

The Innocence Project revealed a significant number of mistakes caused by subjective judgment, leading to innocent convictions (Innocence Project, 2022). Based on these findings, legal research made recommendations for reducing the risk of mistakes. These recommendations assume that mistakes are the result of a lack of caution.

The recommendations were inconsistent, however. They failed to agree on whether predetermined rules or free proofs should guide the legal fact-finding process (Stein, 1996); whether internal belief, coherence, value balancing, or probability should guide the discretion of fact-finders (Sullivan, 2019; Acharya, 2020); and which safeguards should guide the risk allocation: due process, protecting human rights, or credibility (Edmond, 2015). These recommendations made only a limited contribution in preventing wrongful convictions (Raymond, 2001), suggesting that the main reason for legal mistakes was not a lack of caution.

## The Legal Truth

The legal fact-finding process is based on witness reports about sources of information, such as eyewitness testimonies and scientific measurements. Although the fact finders examine the validity of witness testimonies based on reports of various interviewing techniques, such as cognitive interviews, or based on scientific department reports, and the legal fact-finding model relies mainly on estimations and risk management. Fact finders hear witness testimonies, assess their validity based on their perceptions, and decide what has happened in reality (Granot et al., 2018). Although fact finders also consider the relationship between crime scene findings and the witnesses' reports, they examine only whether these correlate. They use the synthesis of evidence, logic, life experience, and the rules of evidence to determine whether to rely on the testimony (Cao and Zhang, 2017). Finally, their decisions are guided by confidence in the coherence of the information (Spottswood, 2013).

Court rulings show that making decisions based on these guiding principles may be problematic because (a) the correlations might be random, being based on statistics with low probability (State V. Morrow, 2005); (b) the justifications might contradict scientific research findings, as in the case of a credible eyewitness identification under problematic viewing conditions and a delayed lineup (Neil V. Biggers, 1972); (c) evidence synthesis might point to a certain conclusion even if a contradictory hypothesis cannot be discarded (Turner V. United States, 2015) or if crucial information needed for validation, such as the possible error rate, is missing (State V. Anderson, 1994; United States V. Mitchel, 2004) or not examined at all (People V. Negron, 2012).

Brain science research may reveal the reasons why deciding based on these principles can be problematic, showing that prior knowledge unconsciously biases perception and can lead to false confidence about reality even if the pieces of information are sparse or inaccurate. Thus, deciding based on the confidence level regarding evidence coherence might diverge from reality because it may be based on the witnesses' and fact finders' prior knowledge rather than on evidence (Smith and Studebaker, 1996; Albright, 2017). Several psychological research findings regarding cognitive biases can also explain why deciding based on subjective perception regarding what happened at a crime scene could lead to mistakes. First, fact finders may be affected by tunnel vision (Findley and Scott, 2006). Tunnel vision is the tendency of actors in the criminal justice system to use heuristics and shortcuts to filter evidence selectively and build a case for a suspect's conviction, ignoring or suppressing evidence that points to the suspect's innocence. Tunnel vision motivates investigators, prosecutors, and judges to attribute importance and relevance to information that supports their original conclusion, while information inconsistent with the presumption of guilt is easily overlooked, dismissed as irrelevant, or considered unreliable (Findley, 2012). Second, the fact finders' decisions may also rely on several aspects of false judgments, e.g., about the plausibility of the testimony (Hartwig and Bond, 2011; Elaad, 2019). Finally, other biases, such as confirmation bias, hindsight bias, and outcome bias are frequently observed in the criminal justice system (Findley, 2012). For example, confirmation bias is the favoring of information that confirms an individual's preconceptions or hypotheses independently of the truth of the information. Forensic findings can be also interpreted in a biased way.

By contrast, scientific research suggests that the validity of a hypothesis must be examined based on experiments and observations, and the results compared with nature because the hypothesis may prove to be wrong (Chamberlin, 1897; Platt, 1964; Feynman et al., 2015).

This perspective examines the possibility of externally validating the legal truth using witness memories and physical crime scene traces based on combined life science methods. Note, however, that intentions play a role in the legal truth and that it is difficult to state that there is an absolute truth. Therefore, external validity measurements of the legal truth are limited by resources and constraints, and concern mainly the identification of the perpetrator. Nevertheless, properly identifying the perpetrator is crucial for differentiating between innocence and guilt.

## Traces From Past Events

The scientific method is based on careful observations and measurements. Hypotheses are always compared with data and are either confirmed or refuted. The legal fact-finding process concerns a particular past event reported by witnesses. Thus, observations of past events and experiments about them are conceptually challenging.

Scientific research has addressed this measurement problem in other fields of research, such as astronomy and archeology, where it is also impossible to conduct experiments or make direct observations of past reality. Nevertheless, hypotheses are being compared with traces of reality based on scientific principles. Recent advances in these fields have shown high accuracy and precision based on minimal evidence from the examined reality. Examples include learning about the early days of the solar system based on carbonaceous asteroid samples (Clark, 2022) or about the structure of the universe based on multi-scale feedback from black holes using low-frequency radio observations (McKinley et al., 2022).

A similar approach may be applied in the legal field because reality leaves traces both at the crime scene and in the witnesses' memories. This approach is already used in forensic thinking. Locard noted that “every contact leaves a trace” (Locard, 1920). But traces from past events can have flaws: memories change constantly, and the traces that reality leaves may not be correct. Furthermore, many of our memories do not last long, and a forgetting process starts immediately. Nevertheless, their connection to the examined reality may be stronger than eyewitness reports and the fact finders' subjective perception. The legal system has already examined traces from the crime scene based on scientific evidence and has compared them with witness testimonies. However, guided by the Daubert trilogy (the legal test used to examine the validity of the scientific norm), the legal fact finder assumes that following a scientific norm proves the conclusion as an absolute fact (Caudill and LaRue, 2003; Faigman et al., 2016; Nir and Liu, 2021). Since the legal system seeks absolute facts, assumptions are needed to apply the scientific method to the legal context. But the legal validity test does not take into account the effect of these assumptions on the strength and validity of the conclusion, so the scientific method is not properly applied by the legal system (Kaye, 2010; Christensen et al., 2014; Carr et al., 2020). The best example is latent fingerprint comparison, where fact finders examine the scientific norm assuming that the experts can evaluate the quality of the fingerprint and determine, in a dichotomous manner, whether there is an absolute match (United States V. Mitchel, 2004). However, scientific research shows that there are many diverging assumptions used by experts in assessing the quality of latent fingerprints because by nature they are small, unclear, often overlap other fingerprints, etc. These assumptions, different for each expert, are about whether it is a single fingerprint, whether marks are related to the fingerprint or the background, etc. (Ulery et al., 2011). Although these assumptions may affect the strength and validity of the expert's conclusion, the courts currently fail to examine their possible influences. Currently, the legal system does not treat traces from the crime scene as external validity.

## Sensitivity to the Perpetrator's Identification From Traces of Past Events Based on life Sciences

In science, accuracy is measured by sensitivity and specificity. Sensitivity refers to true positives (e.g., how many sick people are correctly identified), whereas specificity refers to true negatives (e.g., how many healthy people are correctly identified as being sick).

Science aims to consolidate a general understanding of nature. To this end, scientists make observations, conduct experiments, gather information, and advance research based on accurate measurements (Ayala, 2009). Unlike the legal system, science seeks mainly to obtain a wider universal understanding rather than measure individual occurrences.

Still, sensitivity to the perpetrator's identification based on past events can be guided by the principles of life sciences, such as biology and brain sciences. The best example is genetics. The DNA molecules encode the biological information of each individual. This specificity follows from the laws of inheritance, proven by many observations and experiments (Bolinska, 2018).

Another promising direction to increase sensitivity in identifying the perpetrator is measuring memory traces. Human beings memorize sensory information about the surrounding world. A basic survival mechanism is noticing changes in the environment. When a change occurs and is perceived, irregularities attract attention (Huettel and McCarthy, 2004). These are memorized by different groups of neurons and brain areas at different levels. Subconscious brain memories are located in low-level regions of the brain and are more specific, whereas conscious memories are located in high-level regions and are more general (Lacy and Stark, 2013). The reason for the generality of conscious memories is that they are consolidated based on integration from many brain regions, following the “all or nothing” principle. This principle means that the neural signal is transmitted only if the signal strength is strong enough above the neural noise (Bachmann, 2013). Thus, conscious memories are less specific than subconscious ones.

Perception research has studied the underlying mechanisms of consciousness, revealing measurable subconscious neural activities (Cohen et al., 2016). No-report paradigms (neurophysiological measurements that bypass the need to rely on the observer's report) have been used to measure neural activity. These paradigms are based on neurophysiological activity measurements, such as brain waves or eye movements, obviating the need to rely on the observers' report and offering a more objective measurement tool with high accuracy rates (Tsuchiya et al., 2015; Arzi et al., 2020). Research methods have been developed to accurately measure involuntary physiological and neurophysiological responses and to differentiate between responses to expected and unexpected stimuli by neural biomarkers, such as the P300 brain wave or prolonged micro-saccadic eye movement inhibition (Rosenfeld, 2018). Recent advances in these methods reveal that some brain responses are sensitive to familiarity and these responses can be accurately differentiated when presented on the fringe of awareness. There has been some evidence of a possible linkage between these responses and working memory, including fast crude recognition of familiarities in the early time window, which may be linked to subconscious memories (Moutard et al., 2015; Hacker et al., 2019; Rosenzweig and Bonneh, 2019).

A recent report of a concealed information test using the rapid serial visual presentation (RSVP method) on the fringe of awareness showed high accuracy of P300 brain wave responses to familiar faces, compared with observer reports, even if the observers tried to conceal their knowledge (Bowman et al., 2014). Other research on facial image identification found that even if the observer's reports were mistaken, their eye movement measurements revealed their recognition of facial images (Hannula et al., 2012). Similar results were found in functional MRI (fMRI) studies (Ronzon-Gonzalez et al., 2019). These methods may be used to measure memory traces by measuring neurophysiological responses during a computer lineup in a serial presentation of facial images to the observers to reveal responses to familiarity. Memory measurement has been known for decades, but until recently it has not been used in practice, except in a few anecdotal cases (such as Harrington V. State, 2003). The reason is mainly that their implementation is complicated and not without flaws, and that memory changes constantly and is not being scientifically considered a reliable source of information. Despite these limitations, scientific research shows that memory measurements are frequently more accurate than the observer's report, and can add relevant specific information because it is little influenced by cognitive biases (Tsuchiya et al., 2015; Albright, 2017; Arzi et al., 2020). In the legal system, eyewitness testimony is considered to be solid direct evidence that can confirm perpetrators' identification straightforwardly (Damaška, 1975; Greenstein, 2009). There is a current tendency in the US courts to admit neurophysiological measurements under the Daubert standard, owing to long-standing empirical data showing that the legal concerns about possible fact-finder biases are not as significant as previously believed (Kehl et al., 2017; Roth, 2017; Shen et al., 2017). This tendency is consistent with the US National Academy Report from 2014 (National Research Council, 2014; Albright, 2017), suggesting the use of several scientific techniques to validate the perpetrator's identification by eyewitnesses, which are less sensitive to potential biases, such as sequential lineups. These recommendations were recently adopted by several US states (Albright and Rakoff, 2020). The accuracy of objective memory measurements has been confirmed by a growing body of research findings (Rosenfeld, 2018). Some countries have considered and declined suggestions of using objective memory measurements in the criminal process, or limited the use of similar methods for suspects but not for witnesses because of unclear accuracy rates and legal concerns about human rights, such as the right to due process. Another reason concerned the potential bias of the legal fact finders, which might lead to an increased tendency to incriminate the defendants based on these measurements. But these concerns are less significant given the growing body of research findings demonstrating high accuracy rates, and the legal safeguards developed to minimize risks of infringing human rights, such as the right to due process (Pardo, 2006; Meixner, 2015). This perspective suggests using novel neurophysiological measurements in a new manner that answers many of these concerns. (A) Measuring involuntary eye movements by an external camera is minimally invasive and appropriate for legal work. Involuntary eye movements are minimally influenced by potential biases and can provide relevant subconscious information about specific low-level memories. Although this information is only partial and has limitations, this perspective suggests that it should not be ignored. (B) Can be used as a warning of mismatches between the observers' report and the objective measurements, similarly to using DNA evidence for the Innocence Project. (C) Legal safeguards can be adopted to answer the objections, e.g., the right to refuse the examination and the need to declare the limitations of the measurements (Meixner, 2018). Traditional biological principles, such as heredity, may also be used for identification, but their specificity, which is currently based on assumptions, should be based on measurement as far as possible.

## Specificity by Combined Methods

Accurate measurements play a critical role in science. By contrast, in the legal system, guidance is based on estimation. Yet ironically, the legal system determines absolute “facts” (Dworkin, 1985), therefore, legal fact finders seek absolute scientific answers, side-stepping the kind of measurements science could easily provide (Saks and Koehler, 2008). The estimated accuracy of traces from past events is limited to the kind of specificity science can provide, rather than the absolute identification of the perpetrator, which criminal law seeks. Although this type of external validity measurement has limitations (Pompanon et al., 2005), it offers a better connection between legal fact finding and the examined reality than what is currently used.

Many forensic methods reach conclusions based on assumptions about the legal applicability of findings. For example, a basic assumption of a fingerprint comparison is that experts can determine a match based on their senses. The specificity of fingerprint comparison may be improved by combining measurement and psychophysics (Cole, 2008). As illustrated by this example, combining several scientific methods can increase the specificity of the measurement. Recent studies have shown how combining several scientific methods can increase the specificity of measurements of memory and physical traces of past events. A recent example is the use of involuntary eye movements to reveal concealed information that exists on the fringe of awareness. The traditional concealed information test assumes that the neural responses to unexpected stimuli derive from familiarity. Yet, the neural system responds differently to stimuli presented on the fringe of awareness. Thus, the two methods may be combined to measure responses to surprising stimuli by isolating responses to familiar ones (Alsufyani et al., 2019; Rosenzweig and Bonneh, 2020). Another example is assessing the accuracy of eyewitness identification in a lineup using psychophysics. This method is adjusted to the way perception operates by using relative judgment in a serial presentation of pictures, such as the suspect, and calculating the probability of correct identification (Gepshtein et al., 2020).

Forensic DNA comparison demands DNA amplification, a process that generates byproducts called stutter. Traditional DNA comparison methods assume that DNA alleles and stutter can be differentiated by a standard procedure. A recent DNA study showed that temperature change can differentiate between DNA allele and stutter with greater accuracy than the assumed standard (Seo et al., 2014). These methods increase specificity and show how combining several techniques can boost the accuracy of legal fact finding by external measurement.

## External Validation of the Legal Truth

Revealing what has happened at a past event and the need to properly identify the perpetrator requires external validity measurements. Scientific quantification of traces from past events can create external validity measures. The legal system gathers information from witnesses. Measurement of neurophysiological responses, such as eye movements (no-report paradigms), seems tailor-made for the external validation of witness reports. Combining several scientific methods can increase the specificity of crime scene traces, making it possible to replace the use of assumptions. A recent study showed that in cases of sexual assault, DNA analysis detected wrongfully accused individuals mistakenly identified by eyewitnesses (Wickenheiser, 2021).

Differences between the legal and scientific fact-finding processes suggest that it is necessary to change our way of thinking about legal fact finding. On one hand, legal fact finders should learn from science the meaning and importance of external validity measurements and acknowledge its limitations. To this end, in addition to the Daubert guidelines, they should examine how the adaptation of the scientific norm affects the legal system. They should consider the influence of the assumptions needed for individualization of the scientific norm on the strength and validity of the conclusion. On the other hand, scientific experts should understand the need to quantify the effect of adopting the scientific norm to the legal framework by assessing the influences of the assumptions on the strength and validity of the conclusion. This could be accomplished by increasing possible error rates and emphasizing limitations in their measurements.

Further research is needed to increase the specificity of the scientific methods used in legal fact finding, taking into account current research limitations. Acknowledging the fact that discretion and risk management guide the legal fact-finding process, it seems that the use of crime scene traces as an alert for fact finders when assessing the witnesses' impressions may comply with the legal way of thinking and with the need to achieve many diverging goals and reach a decision within a certain time frame.

## Summary and Conclusion

In almost every criminal case, traces from the crime scene are present in witnesses' memories and physical findings. These may be used for external validity measurements in the legal fact-finding process. Recent methods developed to measure involuntary brain responses to familiarity may be preferable to observer reports because they are based on fast crude recognition of specific familiar features by subconscious memories, unlike the conscious memories, which combine sensual information with prior knowledge.

Involuntary brain responses may be used to externally validate eyewitness testimonies in perpetrator identification at lineups. Combining several scientific methods can increase the specificity of physical trace measurements, which can also be used for external validation. This has the potential to change the current legal practice, which examines only the validity of the scientific norm but ignores its adaptation to the legal framework, although the norm is often changed within the legal framework because of legal assumptions that affect the strength and validity of the conclusions.

External validity measurements are crucial for the legal fact-finding process to reveal the truth. They may be used as alerts for the legal fact finder to help prevent wrongful convictions. Additional research is needed to suggest adjustable scientific measurements and proper legal frameworks.

## Data Availability Statement

The original contributions presented in the study are included in the article/supplementary material, further inquiries can be directed to the corresponding author/s.

## Author Contributions

The author confirms being the sole contributor of this work and has approved it for publication.

## Conflict of Interest

GR obtained with Prof. Yoram S. Bonneh a U.S patent no Us10568557 that is related to one of the methods mentioned in one reference of the perspective.

## Publisher's Note

All claims expressed in this article are solely those of the authors and do not necessarily represent those of their affiliated organizations, or those of the publisher, the editors and the reviewers. Any product that may be evaluated in this article, or claim that may be made by its manufacturer, is not guaranteed or endorsed by the publisher.
